# Effect of low‐pressure cold plasma processing on decontamination and quality attributes of Saffron (*Crocus sativus* L.)

**DOI:** 10.1002/fsn3.2824

**Published:** 2022-03-14

**Authors:** Haleh Darvish, Yousef Ramezan, Mohammad Reza Khani, Amir Kamkari

**Affiliations:** ^1^ Department of Food Science and Technology Faculty of Pharmacy Tehran Medical Sciences Islamic Azad University Tehran Iran; ^2^ Nutrition & Food Sciences Research Center Tehran Medical Sciences Islamic Azad University Tehran Iran; ^3^ Laser and Plasma Research Institute Shahid Beheshti University Tehran Iran; ^4^ Department of Food Engineering Faculty of Agriculture University of Tabriz Tabriz Iran

**Keywords:** crocin, low‐pressure cold plasma, picrocrocin, saffron, safranal

## Abstract

This study investigated the microbial decontamination of saffron using the low‐pressure cold plasma (LPCP) technology. Therefore, other quality characteristics of saffron that create the color, taste, and aroma have also been studied. The highest microbial log reduction was observed at 110 W for 30 min. Total viable count (TVC), *coliforms*, molds, and yeasts log reduction were equal to 3.52, 4.62, 2.38, and 4.12 log CFU (colony‐forming units)/g, respectively. The lowest decimal reduction times (D‐values) were observed at 110 W, which were 9.01, 3.29, 4.17, and 8.93 min for TVC, *coliforms*, molds, and yeasts. LPCP treatment caused a significant increase in the product's color parameters (L*, a*, b*, ΔE, chroma, and hue angle). The results indicated that the LPCP darkened the treated stigma's color. Also, it reduced picrocrocin, safranal, and crocin in treated samples compared to the untreated control sample (*p* < .05). However, after examining these metabolites and comparing them with saffron‐related ISO standards, all treated and control samples were good.

## Graphical abstract

Saffron was treated by radiofrequency (RF) low‐pressure cold plasma (LPCP). Higher input powers and exposure times efficiently reduced the microbial load. All saffron color parameters significantly increased in treated samples, and LPCP significantly decreased saffron's҆ secondary metabolites.

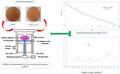

## 1 INTRODUCTION

Nowadays, spices play a crucial role in the human diet all over the world. Herbs and spices are both categorized as plants. Herbs are the green parts of a plant while spices are seeds, bark, and underground stems that ameliorate flavor, color, giving fragrant smell, or preserving foods by their defensive component such as antioxidant and antimicrobial properties. Spices are also rich in phytochemical compounds to protect them against insects called host resistance activity (Caranta & Dogimont, 2008).

Saffron (also known as red gold) is the most expensive spice worldwide, which contains dried red stigmas of an autumnal cultivated flower belonging to the *Iridaceae* family, *Crocus* genus and *C. sativus* species (Lozano et al., 2020). Since saffron is adapted to arid and semiarid climate, it has been extensively cultivated in Spain, India, Afghanistan, Greece, Morocco, and notably Iran, which has the largest cultivated area and the most obtained product. The total amount of Iran's saffron production in 2018 was around 400 tons (Abolhassani et al., 2020).

Saffron contains high amounts of carotenoid pigments that among them crocin (digentiobiosyl derivative of the apocarotenoid crocetin), picrocrocin (β‐D‐glucoside of hydroxysafranal), and safranal (the bio‐oxidative cleavage product of zeaxanthin) are three main secondary metabolites in Saffron's stigma that are, respectively, responsible for color, bitter taste, and aroma. It must be mentioned that they are all derived from the hydrolysis of zeaxanthin (Moratalla‐López et al., 2021; Sarfarazi et al., 2019; Zhang et al., 2019).

Nowadays, saffron is used and traded because of its medical, cosmetic, coloring, and flavoring properties. Saffron poses a positive effect on neurological disorder, antioxidant ability, anticancer (a particularly malignant tumor of the uterus), antimicrobial (due to azafrines 1 and 2), and anti‐inflammatory agent (Martí et al., 2020). Also, a positive effect on the redness of the skin is caused by an increased blood flow (known as erythema) in addition to insomnia and anti‐atherosclerotic (buildup of fats), which is the result of crocin and crocetin (Huang et al., 2015; Mzabri et al., 2019; Pandita, 2021; Vuolo et al., 2019).

If proper care is not taken, every step in the supply chain can affect spice quality and account for food safety issues. During harvesting, processing, storage, and handling, improper practices and conditions significantly affect spice quality (Thanushree et al., 2019).

The spices frequently get contaminated with *Bacillus cereus, Bacillus coagulans, Bacillus polymyxa, Bacillus subtilis*, *Clostridium perfringens*, and *Salmonella* spp., *Escherichia coli* (*E. coli) O157:H7*, *fecal streptococci* isolated, fungus‐like *Aspergillus flavus*, *Aspergillus niger*, *Aspergillus nidulans*, *Penicillium* spp., and *Rhizopus* spp. Dried spices with low water activity are often susceptible to fungal contamination, and their safety remains a challenge for processors (Misra et al., 2019). This microorganism can cause foodborne diseases if the spices are improperly dried, moistened during storage, or rehydrated by incorporating into formulations to get consumed so they should be prepared by a suitable method (Hertwig, Reineke, Ehlbeck, Erdoğdu, et al., 2015; Pinkas & Keller, 2014; Yogendrarajah et al., 2016).

Common technologies for microbial disinfection of spices are gamma irradiation, superheated steam treatment, and fumigation with ethylene oxide and propylene oxide. However, all mentioned methods have some drawbacks and limitations (Hertwig, Reineke, Ehlbeck, Erdoğdu, et al., 2015; Hertwig, Reineke, Ehlbeck, Knorr, et al., 2015). Gamma irradiation has been shown to be successfully decontaminated spices. Irradiation with gamma‐rays can only be applied in authorized facilities and controlled doses. Moreover, this process has low consumer acceptance in the European Union (EU) due to the consumers’ concerns (Kim et al., 2014). Steaming in spices with a high microbial load is not recommended because it has adverse effects on color, flavor, and nutritional properties (Bang et al., 2020). Fumigation with ethylene oxide and propylene oxide is widely used in the USA, but banned in many countries because it can lead to carcinogenic by‐products. Considering the above drawbacks and issues and increasing consumers’ demands for safe and high‐quality foods, it becomes necessary to develop new alternative decontamination processes. The application of cold plasma can be an alternative technology for the decontamination of herbs and spices (Hertwig et al., 2018; Kim et al., 2017).

Cold plasma is defined as a partially ionized gas comprising highly reactive species (reactive oxygen and nitrogen species), ions with a negative or positive charge, free radicals, electrons, gas atoms, and molecules, and electromagnetic radiation (photons) quantum at room temperature (Afshar et al., 2022; Gholamazad et al., 2022).

While a few studies have focused on the effects of vacuum base radiofrequency (RF) low‐pressure cold plasma (LPCP) on spices, this study aimed to investigate the efficiency of LPCP generated by RF on microbial decontamination, color, and secondary metabolites of saffron.

## 2 MATERIALS AND METHODS

Dried saffron was purchased from Qaen (South Khorasan Province, Iran), the center of saffron production in Iran. Then, saffron samples were weighed in sterile packages and kept inside them until the experiments were performed. All chemicals and culture media used in the tests were purchased from Merck (Germany).

### 2.1 Plasma reactor and sample treatment

RF LPCP reactor with a 13.6 MHz frequency was used as a parallel‐plate capacitively coupled configuration (Plasma Clean‐Satia Company, Tehran, Iran). The system was equipped with rotary and Roots vacuum pumps contained in a stainless steel chamber with a 27 cm diameter and a water‐cooled electrode with a 11 cm diameter.

**FIGURE 1:**
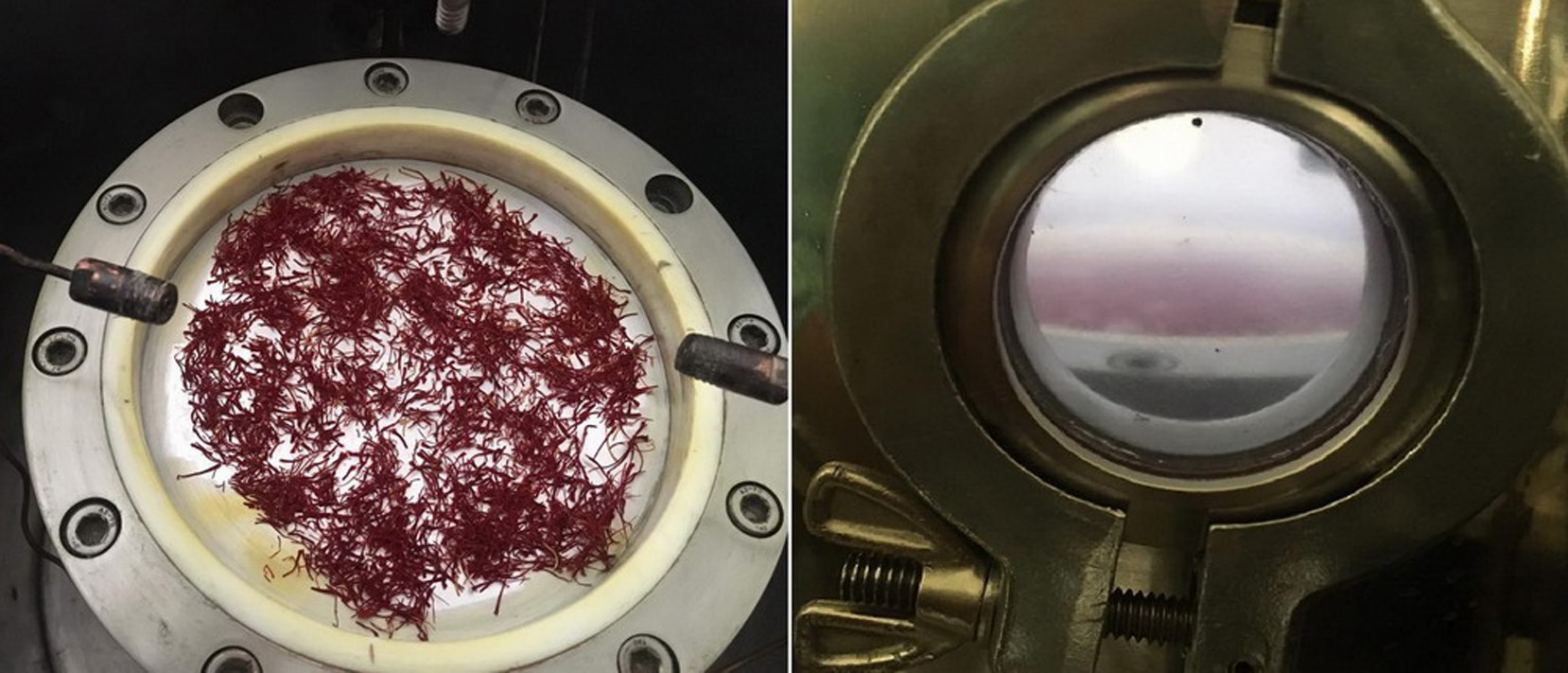
Inside and outside of radiofrequency (RF) low‐pressure cold plasma chamber during saffron treatment

Before the test, the device's inner chamber was decontaminated with 70% ethanol and rinsed with sterile distilled water, and dried naturally in the biohazard hood. The samples were then placed inside the machine chamber (8 g of saffron), and the chamber was closed (Figure 1).

The rotary mechanical pump, which is usually the starting point for creating a vacuum in the system, was then turned on. After the chamber's pressure reached less than 13.33 Pa, the Roots rotary mechanical pump supported by the rotary pump was switched on. The pressure was allowed to reach the lowest value inside the chamber.

After reducing the desired pressure, pure oxygen gas (99.99%) was introduced into the chamber with a flow rate of 20 SCCM (standard cc/min). A cold tap was opened to prevent the temperature of the chamber and the treated samples from rising. The device was turned on, and the samples were treated with the powers considered in this research at 70, 90, and 110 W for 5, 10, 15, and 30 min. The plasma was produced by injecting reactive gas, which is oxygen here, between two parallel electrodes with an 8‐cm discharge distance, a ground electrode, and an electrode connected to an RF generator, and capacitive coupling between the two electrodes converted the gas to plasma. After the samples’ treatment, the device was turned off, and the treated samples were examined for microbial load reduction and quality characteristics compared to the control sample. The schematic of the plasma device and its details are fully described in our previous study (Kashfi et al., 2020).

### 2.2 Microbial enumeration

The total viable count (TVC) was determined according to ISO 4833‐1:2013, and *coliforms* count test to ISO 4832:2006, *Escherichia coli* count according to ISO method 7251:2005, and molds and yeasts counts were performed according to the ISO method 21527‐2:2008 (ISO, 2005, 2006, 2008, 2013).

### 2.3 D‐value's measurement

D‐value is defined as the time necessary for a 90% reduction in the microbial population. Alternatively, the D‐value is the time required for one log‐cycle reduction in the population of microorganisms. Equations 1 and 2 can be used to calculate the D‐value. Equation 2 is the negative inverse of the slope of the survivor curve. Results were expressed in min (Singh & Heldman, 2001).

(1) $$D‐\mathrm{value}\;=\frac{t}{\log N_{0}‐\log N}$$

(2) $$D‐\text{value}=‐\left(\frac{1}{\text{slope}}\right)$$

*t* is the processing time; *N*_0_ is the initial microbial population; and *N* is the final microbial population.

### 2.4 Color measurement

Colorimetry was performed by HunterLab colorimeter (CR‐400; Konica Minolta Censing Inc., Osaka, Japan) and the parameters L* (brightness/darkness), a* (redness/greenness), and b* (yellowness/blueness) were measured. From these three parameters, the total color difference index (∆E) of the treated samples and the untreated sample, which is used here as a reference, was calculated using Equation 3. Chroma (which is the saturation index and color intensity of the sample) and hue angle were also calculated by Equations 4 and 5 (Yong et al., 2017).

(3) $$\Delta E=\sqrt{{\Delta L\;}^{2}+{\Delta a\;}^{2}+{\Delta b\;}^{2}}$$

(4) $$\mathrm{Chroma}\;=\sqrt{a^{2}+b^{2}}$$

(5) $$\text{Hue}\;\text{angle}=\tan^{‐1}\left(b/a\right)$$

### 2.5 Saffron secondary metabolites analysis

The levels of secondary saffron metabolites (picrocrocin, safranal, and crocin) were measured according to ISO 3632‐2 (2010) using the Cary 100 UV‐Visible Spectrophotometer (Agilent Technologies, the USA). For this purpose, 500 mg of each sample was ground, and after transfer to a 1000 ml volume balloon, 900 ml of distilled water was added to it. It was then mixed with a magnetic stirrer for 1 h at a speed of 1000 rpm. The solution's volume was increased to 1000 ml with distilled water and stirred again to obtain a uniform solution. Twenty milliliters of the resulting solution was brought to a volume of 200 ml. The solution was filtered, and after obtaining a clear solution, the amount of absorption was recorded at wavelengths of 200 to 700 nm relative to the reference (distilled water). The results are obtained by a direct reading of the specific absorbance at three wavelengths, as follows:

Absorbance at about 257 nm (λ max of picrocrocin), 330 nm (λ max of safranal), and 440 nm (λ max of crocin) (ISO, 2010).

(6) $$A_{1\;\mathrm{cm}\;}^{1\%}=\;\frac{D\;\times 10000}{m\;\times (100‐w_{\mathrm{MV}})}$$

$A_{1\;cm\;}^{1\%}$ is the absorbance at the maximum wavelength of secondary saffron metabolites for a 1 g/100 ml solution of test sample using a 1‐cm quartz cell; D is the specific absorbance; m is the mass, in grams, of the test portion; *w*_MV_ is the moisture and volatile matter content, expressed as a percentage mass fraction, of the sample.

### 2.6 Statistical analysis

All the experiments were performed in triplicate orders. The data were analyzed with Minitab 16 software. The effect of the radiofrequency LPCP treatment was determined through a one‐way analysis of variance (ANOVA), and the differences between the means were determined by Tukey's test at (*p* < .05).

## 3 RESULTS AND DISCUSSION

### 3.1 Microbial decontamination

The results showed that the treatment of saffron stigmas with LPCP successfully reduced the microbial load of saffron (*p <* .05). As the highest power–time combination used in this study, 110 W for 30 min reduced 3.5 log CFU/g in TVC and 4.12 log CFU/g in the number of yeasts (Table 1). Some studies have suggested that higher power and longer plasma treatment times produce more active species, leading to more disinfection (Kim et al., 2017; Song et al., 2015). Also, molds and *coliforms* have been completely disabled. In the last two cases, according to the results, it was found that the amount of complete inactivation of molds with lower powers also occurred. For example, molds were completely inactivated at the lowest power used in the present study (70 W, 30 min) (Table 1). The results were obtained about reducing the microbial population in line with the national standards of Iran, microbiology of saffron‐specifications and test methods (INSO, 2018). *E. coli* was not observed in any of the LPCP‐treated samples and control.

**TABLE 1:** Effects of different low‐pressure cold plasma (LPCP) powers and exposure times on total viable count, *coliforms*, molds, and yeasts (log CFU (colony‐forming units)/g) in saffron

Input power (W)	Plasma exposure time (min)	Total viable count (log CFU/g)	*Coliforms* (log CFU/g)	Molds (log CFU/g)	Yeasts (log CFU/g)
Control	0	4.67 ± 0.03^a^	4.62 ± 0.03^a^	2.38 ± 0.00^a^	5.27 ± 0.03^a^
70 W	5	4.62 ± 0.03^a^	4.50 ± 0.06^a^	1.80 ± 0.01^a^	4.95 ± 0.00^b^
	10	4.38 ± 0.12^a^	4.23 ± 0.08^a^	1.50 ± 0.01^ab^	4.73 ± 0.05^bc^
	15	3.92 ± 0.10^b^	4.06 ± 0.08^ab^	0.50 ± 0.70^c^	4.53 ± 0.08^c^
	30	3.45 ± 0.21^cd^	1.50 ± 2.12^bcde^	0.00 ± 0.00^c^	2.92 ± 0.10^g^
90 W	5	4.38 ± 0.12^a^	4.45 ± 0.07^abc^	0.65 ± 0.92^bc^	3.97 ± 0.03^d^
	10	3.84 ± 0.21^bc^	3.53 ± 0.33^abc^	0.50 ± 0.70^c^	3.82 ± 0.18^d^
	15	2.95 ± 0.06^ef^	2.84 ± 0.21^abcd^	0.00 ± 0.00^c^	3.53 ± 0.08^e^
	30	2.58 ± 0.15^fg^	0.65 ± 0.92^de^	0.00 ± 0.00^c^	2.47 ± 0.00^h^
110 W	5	3.48 ± 0.16^cd^	2.34 ± 0.49^abcde^	0.50 ± 0.70^c^	3.58 ± 0.15^e^
	10	3.36 ± 0.15^de^	1.15 ± 0.21^cde^	0.00 ± 0.00^c^	3.30 ± 0.00^f^
	15	2.34 ± 0.49^g^	0.00 ± 0.00^e^	0.00 ± 0.00^c^	3.08 ± 0.12^fg^
	30	1.15 ± 0.21^h^	0.00 ± 0.00^e^	0.00 ± 0.00^c^	1.15 ± 0.21^i^

Note

Results were expressed as mean ± SD. Different superscript letters in the same column mean significant differences (*p* < .05).

In this study, pure oxygen gas was used for decontamination. Plasma processing produces reactive oxygen species (ROS) such as atomic oxygen O* and molecular oxygen ions O_2_^+^, which are primarily responsible for microbial decontamination because they cause oxidative effects on the microbial outer surface. Excited atoms, free radicals, ions, active species, and ultraviolet (UV) photons interact with microorganisms in various ways and cause decontamination. Other mechanisms, such as lipid peroxidation and protein modulation, and bacterial apoptosis, can be mentioned (Attri et al., 2015; Liao et al., 2019).

The bombardment of microorganisms by energetic ions causes perforation and rupture of the protective wall, caused by erosion and etching of the cell wall. In addition, the accumulation of charged particles on the microbial cell wall causes electrostatic stress, which overcomes the tensile strength of the cell wall, leading to morphological changes in the cell wall (Bourke et al., 2017).

In a study conducted by Akbarian et al. (2019), saffron was decontaminated by dielectric barrier discharge (DBD) cold plasma method. In this research, cold plasma was produced by two types of gas, including nitrogen and normal air. The results indicated that the disinfection effects of nitrogen plasma were lower than those of air plasma. Increasing plasma exposure time had a significant effect on microbial reduction. The maximum microbial reduction was observed at 12 min and 18 kV (Akbarian et al., 2019).

Previous studies indicated that cold plasma is regarded as a promising nonthermal technology for spice decontamination, without significant changes in other quality parameters of these products. Bang et al. (2020) studied black peppercorns’ decontamination by simultaneous treatment with cold plasma and ultraviolet C. Simultaneous UV‐CP treatment inactivated indigenous bacteria and *Bacillus tequilensis* spores by 3.4 log CFU/g. In comparison, the sum of individual UV and CP treatments’ inactivation rates was 2.7 log CFU/g. Also, the UV‐CP treatment did not alter black peppercorns’ color (Bang et al., 2020). In another study, Kim, Oh, et al. (2017) investigated microwave cold plasma treatment's effects on *Aspergillus brasiliensis* inoculated into onion powder. The results indicated that plasma treatment following vacuum drying of the powder exhibited significant fungal reduction (1.5 log spores/cm^2^) compared to that after hot air drying (0.7 log spores/cm^2^) (Kim, Oh, et al., 2017).

Similar to our study, Hertwig, Reineke, Ehlbeck, Erdoğdu, et al. (2015), Hertwig, Reineke, Ehlbeck, Knorr, et al. (2015)reported that plasma treatment for 5 min completely inactivated yeast and mold on black pepper seeds (Hertwig, Reineke, Ehlbeck, Knorr, et al., 2015).

### 3.2 D‐values

The first‐order kinetics model is commonly used to determine the decimal reduction time in thermal and nonthermal processes. In this study, to obtain D‐values, a semilogarithmic graph using log N0 (CFU/g) versus plasma application times was used (Kim et al., 2020). The results showed that D‐values decreased with increasing plasma power and exposure time. The lowest D‐values obtained were observed at 110 W, which were 9.01, 3.29, 4.17, and 8.93 min for TVC, *coliforms*, molds, and yeasts (Table 2).

**TABLE 2:** Decimal reduction times (D‐values) for different low‐pressure cold plasma (LPCP) powers on total viable count, *coliforms*, molds, and yeasts in saffron

Input power (W)	Total viable count	*Coliforms*	Molds	Yeasts
70 W	23.09 min	15.22 min	12.42 min	12.88 min
90 W	13.24 min	7.87 min	6.41 min	12.13 min
110 W	9.01 min	3.29 min	4.17 min	8.93 min

In a study conducted by Song et al. (2009), D‐values were calculated for *Listeria monocytogenes* treated with atmospheric pressure plasma at 75, 100, 125, and 150 W in sliced cheese and sliced ham. The lowest D‐value in their study is related to the highest power applied at 150 W for sliced cheese (Song et al., 2009). In another study, dried walnut kernels were processed by LPCP treatment using normal air at different powers (20, 30, 40, and 50 W) and times (10, 15, and 20 min). D‐values were calculated, with the maximum reduction observed at 50 W. The lowest D‐values for the TVC, *coliform*, and mold were 19.66, 18.25, and 18.18 min, respectively (Ahangari et al., 2021). The results of these studies are consistent with those of the present study.

### 3.3 Colorimetry

Color can be an important quality parameter for saffron, which is used as a natural food coloring and flavoring agent. Therefore, it is necessary to study the effect of LPCP on the color and taste parameters of this product. The L* parameter indicates brightness or darkness in the product. According to the results, all treated and untreated samples have positive L* values. The highest L* value is related to the treated sample 110 W for 5 min (23.30), which is significantly higher than those of other samples and has more brightness (*p <* .05). Except for samples treated at 70 W, 10 and, 15 min, other samples treated with LPCP had significantly more L* (*p <* .05) than the control sample (21.29) (Table 3).

**TABLE 3:** The L∗, a∗, b∗ color parameters, ΔE, chroma, and hue angle values of the low‐pressure cold plasma (LPCP)‐treated samples and untreated control sample in saffron

Treatments	L*	a*	b*	ΔE	Chroma	Hue angle
Control	21.29 ± 0.01^i^	18.72 ± 0.11^j^	11.98 ± 0.30^h^	0.00 ± 0.00^h^	22.22 ± 0.06^i^	32.62 ± 0.81^fg^
70 W, 5 min	21.87 ± 0.02^e^	20.07 ± 0.15^g^	12.75 ± 0.14^g^	1.64 ± 0.05^g^	23.75 ± 0.05^h^	32.33 ± 1.50^g^
70 W, 10 min	19.95 ± 0.02^k^	22.25 ± 0.06^b^	15.30 ± 0.40^de^	5.40 ± 0.21^cd^	27.01 ± 0.17^cd^	34.80 ± 0.19^ef^
70 W, 15 min	18.95 ± 0.08^l^	22.16 ± 0.19^b^	14.62 ± 0.29^f^	4.93 ± 0.33^d^	26.55 ± 0.32^de^	33.40 ± 0.30^fg^
70 W, 30 min	21.65 ± 0.00^f^	20.17 ± 0.06^a^	15.76 ± 0.26^cde^	4.07 ± 1.26^e^	25.60 ± 0.21^f^	38.00 ± 0.37^bcd^
90 W, 5 min	21.43 ± 0.02^g^	19.60 ± 0.05^h^	15.17 ± 0.15^ef^	3.31 ± 1.13^f^	24.78 ± 0.05^g^	37.73 ± 0.36^cd^
90 W, 10 min	22.98 ± 0.02^b^	21.23 ± 0.04^d^	17.88 ± 0.06^a^	6.63 ± 0.06^a^	27.75 ± 0.07^ab^	40.11 ± 0.04^ab^
90 W, 15 min	22.44 ± 0.07^c^	21.03 ± 0.02^e^	17.33 ± 0.06^ab^	5.94 ± 0.06^b^	27.25 ± 0.02^bc^	39.49 ± 0.13^abc^
90 W, 30 min	22.17 ± 0.04^d^	19.38 ± 0.09^i^	17.34 ± 0.26^ab^	5.47 ± 0.24^c^	25.60 ± 0.10^ef^	41.82 ± 0.57^a^
110 W, 5 min	23.30 ± 0.13^a^	22.45 ± 0.00^a^	17.22 ± 0.41^b^	6.75 ± 0.28^a^	28.30 ± 0.24^a^	37.48 ± 0.67 cd
110 W, 10 min	21.31 ± 0.00^hi^	20.90 ± 0.07^e^	16.23 ± 0.48^c^	4.78 ± 0.39^d^	26.47 ± 0.23^de^	37.82 ± 0.93^bcd^
110 W, 15 min	20.92 ± 0.01^j^	20.54 ± 0.00^f^	15.21 ± 0.03^def^	3.73 ± 0.03^ef^	25.56 ± 0.02^f^	36.55 ± 0.005^de^
110 W, 30 min	21.42 ± 0.00^gh^	21.48 ± 0.04^c^	15.78 ± 0.20 cd	4.63 ± 0.24^d^	26.65 ± 0.08 cd	36.31 ± 0.40^de^

Note

Results were expressed as mean ± SD. Different superscript letters in the same column mean significant differences (*p* < .05).

About the parameters, a* which showed a tendency to redness (+) and greenness (−), all samples treated with LPCP showed a higher ratio of a* to the control sample, and the highest value was again related to the sample treated at 110 W for 5 min (22.45) (*p *< .05). The results showed that saffron stigmas’ treatment with LPCP increased the redness (a*) of the treated samples (Table 3).

The lowest value of b* is related to the control sample (11.98), and the highest value is related to the sample treated at 90 W for 10 min (17.88). The treatment of samples with LPCP increased the samples’ yellowness compared to the control sample (Table 3).

All samples treated with LPCP had a significantly higher total color difference (ΔE) than the control sample (*p <* .05). The largest difference in ΔE compared to the control sample was related to the sample treated at 110 W for 5 min (6.75) (Table 3). This phenomenon can be considered favorable for saffron. Aqueous saffron extracts prepared in boiling water have created more color in the treated samples than in the control sample. This increase in color can be due to various reasons, and two of the most important reasons are as follows. In a study conducted by Hosseini et al. (2018), the effect of cold plasma on the surface of saffron stigmas and scanning electron microscopy (SEM) imaging showed that in treated samples with LPCP, scratches and cracks appeared which are not seen in the control sample. Hence, LPCP‐treated samples required a shorter period to develop its content's color and release it into the boiling water (Hosseini et al., 2018). The primary pigments of saffron are from the carotenoid family. In these compounds, the presence of conjugated double bonds is essential to create a recognizable color. Increasing the number of conjugated double bonds, the higher the absorption maxima (λ_max_), can increase the red color intensity of the product (Khoo et al., 2011). The optical emission spectroscopy (OES) spectrum can be helpful to justify the two cases mentioned before. Because pure oxygen gas is used in this study, the main active oxygen species produced are O_2_^+^ ions and O* radicals, which can create scratches and cracks on the surface of saffron stigmas, and these compounds also have much energy to bring about oxidation of these pigments and change the color intensity in saffron stigmas.

The effect of food processing on carotenoids was studied by other researchers. Naturally, carotenoids predominantly occur in their *all‐trans* configuration, which is the thermodynamically more stable isomer. However, *cis‐isomers* may increase due to the isomerization of the *trans‐isomer* of carotenoids during food processing (Khoo et al., 2011; Schieber & Carle, 2005; Zhang et al., 2016). Some evidence proved that *cis‐isomers* could also isomerize into *all‐trans* isomers when heated and exposed to air (Khoo et al., 2011).

There are three possible pathways that saffron color changed from orange‐red to dark red after LPCP treatment. Photooxidation of pigments due to the combination of light and ROS, which is both available in low‐pressure cold O_2_ plasma. Millard reaction and enzymatic browning by polyphenol oxidase, causing a rapid darkening of plant color, are exposed to the molecular oxygen, but further studies should be done (Kashfi et al., 2020; Kudra & Strumillo, 2014).

The chroma index shows the intensity and saturation of the color. It was found again that LPCP treatment can significantly increase the chroma compared to the control sample. The highest chroma value is related to the treated sample 110 W for 5 min (28.30) (*p *< .05) (Table 3).

Hue angle is an indicator of the color of a food or, in other words, indicates the dominant color of food. Its zero or 360‐degree angle indicates the red color, which changes from zero to 60 degrees when the angle changes from red to yellow. Examination of this angle revealed that all treated and untreated samples are at the end of the red range and the beginning of the yellow range. The treated samples at 90 W (10, 15, and 30 min) with higher b* also have a higher hue angle (Table 3).

### 3.4 Saffron metabolites

The main factors that create the color, taste, and aroma of saffron, namely crocin, picrocrocin, and safranal, are derivatives of zeaxanthin, a carotenoid pigment. The primary color of saffron is a water‐soluble carotenoid called crocin. The taste of saffron is related to the bitter heteroside picrocrocin (β‐d‐glucoside of safranal). The aroma of saffron is due to the presence of colorless terpene essential oil and an oxygenated compound with cinnamon called safranal (2,6,6‐trimethyl‐1,3‐cyclohexadiene‐1‐carboxaldehyde), which is more than 60%–70% essential oil in stigma (Amin & Hosseinzadeh, 2015).

The effect of LPCP on these compounds is given in Table 4. In general, in some samples treated with LPCP compared to the untreated sample, reduction in crocin, picrocrocin, and safranal has been observed. Among all treated samples, 110 W for 10 min had the least effect on these compounds, and there was no significant difference with the control sample (*p >* .05). Although some of these reductions were statistically significant, if all treatments are compared with ISO 3632‐1 (2011), it is clear that saffron quality is well maintained (Table 4). According to this standard, saffron is placed in different categories. In the case of picrocrocin, based on dry weight, if the amount of this compound is at least 70, it is in Category I. In the case of safranal, the minimum amount of this compound is 20, and the maximum amount of this compound is 50. If the amount of crocin based on dry weight is at least 190, it will be in Category I (ISO, 2011). According to the results, the amount of picrocrocin in all samples was higher than 70, the amount of safranal obtained was in the range of 20–50 (31.39–35.17), and the amount of crocin in all samples was higher than 190. Therefore, it can be concluded that although the amount of these compounds was reduced significantly in some treated samples in comparison with the control sample, all samples are in good condition in terms of picrocrocin, safranal, and crocin (Table 4).

**TABLE 4:** Saffron secondary metabolites values of the low‐pressure cold plasma (LPCP)‐treated samples and untreated control sample

Treatment	Picrocrocin (mg/dry weight)	Safranal (mg/dry weight)	Crocin (mg/dry weight)
Control	80.50 ± 0.00^a^	35.17 ± 0.00^a^	230.70 ± 0.81^a^
70 W, 5 min	79.34 ± 0.49^abc^	34.81 ± 0.69 ^ab^	226.26 ± 0.07^b^
70 W, 10 min	77.30 ± 0.28^cde^	33.75 ± 0.17^abcd^	220.65 ± 0.02^de^
70 W, 15 min	73.67 ± 0.18^f^	32.68 ± 0.58^bcde^	207.39 ± 0.58^f^
70 W, 30 min	79.59 ± 0.72^ab^	34.13 ± 0.00^abcd^	224.61 ± 0.69^bc^
90 W, 5 min	80.18 ± 0.96^a^	34.69 ± 0.17^abc^	218.08 ± 0.75^e^
90 W, 10 min	77.40 ± 0.07^cd^	33.65 ± 0.75^abcde^	219.67 ± 0.24^de^
90 W, 15 min	77.19 ± 0.70^de^	32.63 ± 0.04^bcde^	218.90 ± 0.71^e^
90 W, 30 min	77.85 ± 0.35^bcd^	34.56 ± 0.76^abc^	218.01 ± 0.69^e^
110 W, 5 min	75.25 ± 0.00^ef^	32.56 ± 0.07^cde^	222.16 ± 1.48^cd^
110 W, 10 min	81.19 ± 0.40^a^	35.13 ± 0.74^a^	230.72 ± 0.45^a^
110 W, 15 min	73.56 ± 0.66^f^	32.02 ± 1.06^de^	207.87 ± 0.07^f^
110 W, 30 min	77.52 ± 0.66^cd^	31.39 ± 0.68^e^	225.35 ± 0.78^b^

Note

Results were expressed as mean ± SD. Different superscript letters in the same column mean significant differences (*p* < .05).

The effect of gliding arc discharge plasma on the physicochemical properties of saffron was studied by Tabibian et al. (2020). They measured crocin, picrocrocin, and safranal by UV‐vis and HPLC analysis. The results indicated that the quality of all samples could be classified as Category I. They reported that the amount of safranal decreases with increasing the pretreatment time. Because of surface bombardment with cold plasma particles and the reaction of reactive species and UV photons with the surface bonds (causing oxidative breakdown), various statements including pores’ formation, cell wall destruction, and the release of these volatile aldehydes were facilitated. Also, increasing cold plasma exposure time caused a decrease in the amounts of crocin and picrocrocin. The presence of unsaturated bonds in the molecular structure of both compounds made them susceptible to oxidizing agents. The glycosidic linkages of crocin and picrocrocin may be weak or break due to the reaction with active species (free radical), especially at higher pretreatment times (Tabibian et al., 2020). These results are similar to the present study results.

The effect of cold jet plasma on crocin esters and volatile oils of saffron was studied by Amini et al. (2017). Oxygen at two different levels (5, 10%) was added to argon gas at 8 and 12 kV of voltage. The results showed that increasing the input voltage and increasing the amount of added oxygen to Argon gas significantly decreased safranal and crocin esters (*p *< .05) (Amini et al., 2017). In other studies, cold plasma treatment with O_2_ gas reduced these metabolites compared to the control sample (Akbarian et al., 2019; Hosseini et al., 2018). These results are also consistent with the present study results.

Zareena et al. (2001) studied changes in aroma and coloring properties of saffron after gamma irradiation at doses of 2.5 and 5 kGy. They reported that after 5 kg gamma irradiation, the amount of safranal and the amount of isopherone and 4‐ketoisopherone were increased. The concentration of crocetin increased 2.2 times. The crocins decreased by approximately 83% to 89% in the irradiated samples compared to the control samples. They suggested radiation‐induced degradation of crocetin glucosides (Zareena et al., 2001).

Zarghami and Heinz (1971) reported that irradiation with long‐wavelength UV light led to the oxidative breakdown of safranal due to isophorone‐related compounds’ formation. The reduction of safranal compounds after the cold plasma treatment (similar to this study) may be because of the oxidative breakdown of safranal due to the formation of isophorone‐related compounds (Zarghami & Heinz, 1971).

## 4 CONCLUSION

This study investigated the effect of O_2_ LPCP on saffron stigmas. The results showed that increasing the LPCP power and exposure time reduced the microbial load of saffron stigmas. Molds and *coliforms* were completely inactivated. However, this occurred in molds at lower power (70 W) than in *coliforms*, yeasts, and TVC. The D‐values at 70 and 90 W were more significant than 110 W because D‐values are a microbial population decline function. Color measurement results indicated that treatment with LPCP increased all color parameters. ΔE, chroma, and hue angle values also increased. These changes are considered positive in products such as saffron, which can be used as a coloring agent in food products. This is also because it will produce more color in an equal amount. The examination of picrocrocin, safranal, and crocin levels also showed that LPCP treatment reduced these compounds. Among all treated samples, the sample treated at 110 W for 10 min had the best result, and the values of these compounds were similar to the control sample. However, all LPCP‐treated and control samples in terms of picrocrocin, safranal, and crocin were classified according to ISO standards in Category I. The LPCP is a promising method related to the decontamination of saffron, increasing the color intensity and maintaining the saffron quality properties. LPCP, as an emerging technology, can be used in the food industry and replaced by conventional disinfection methods for sensitive foods like spices and aromatic plants.

## CONFLICT OF INTEREST

We wish to confirm that there are no known conflicts of interest associated with this publication and there has been no significant financial support for this work that could have influenced its outcome.

## AUTHOR CONTRIBUTIONS

**Haleh Darvish:** Data curation (equal); Formal analysis (equal); Funding acquisition (equal); Investigation (equal); Resources (equal). **Yousef Ramezan:** Conceptualization (lead); Data curation (lead); Methodology (lead); Project administration (lead); Software (equal); Supervision (equal); Validation (equal); Visualization (equal); Writing – original draft (lead); Writing – review & editing (lead). **Mohammad Reza Khani:** Methodology (equal); Project administration (equal); Resources (equal). **Amir Kamkari:** Writing – original draft (equal); Writing – review & editing (equal).

## Data Availability

The data that support the findings of this study are available from the corresponding author upon reasonable request.
